# Development and external validation of a FISH-clinical nomogram for predicting overall survival in bladder cancer patients after radical cystectomy

**DOI:** 10.1186/s12885-025-14677-w

**Published:** 2025-10-25

**Authors:** Junjiong Zheng, Sihong Lu, Qihang Zhang, Long Zhang, Yi Huang, Jianqiu Kong, Xu Chen, Jie Zhang, Yuhui Yao, Yun Luo, Tianxin Lin

**Affiliations:** 1 Department of Urology, Guangdong Provincial Key Laboratory of Malignant Tumor Epigenetics and Gene Regulation, Guangdong Provincial Clinical Research Center for Urological Diseases, Sun Yat-Sen Memorial Hospital, Sun Yat-Sen University, Guangzhou, People’s Republic of China; 2 Department of Urology, Guangdong Provincial People’s Hospital, Guangdong Academy of Medical Sciences, Guangzhou, People’s Republic of China; 3 Department of Pathology, Sun Yat-sen Memorial Hospital, Sun Yat-sen University, Guangzhou, People’s Republic of China; 4 Department of Urology, the Third Affiliated Hospital of Sun Yat-Sen University, No. 600 Tianhe Road, Guangzhou, People’s Republic of China

**Keywords:** Bladder cancer, Radical cystectomy, Overall survival, Fluorescence in situ hybridization, Nomogram

## Abstract

**Background:**

Bladder cancer has notable heterogeneity. The urine-based fluorescence in situ hybridization (FISH) test can detect bladder cancer noninvasively. In this study, we aimed to construct a nomogram based on FISH results and clinical features (referred to as the FISH–clinical model) to predict the overall survival (OS) of bladder cancer patients following radical cystectomy (RC).

**Methods:**

A total of 261 eligible patients were enrolled for this study. The SYSMH cohort was divided into training (*n* = 138) and internal validation (*n* = 70) sets; the SYSUTH cohort was used for external validation (*n* = 53). Multivariate Cox proportional hazards regression was applied for FISH–clinical model construction, and model performance was evaluated according to analyses of calibration, discrimination ability, and clinical usefulness.

**Results:**

FISH-identified chromosome 7 and 17 aneuploidies correlated significantly with increased pT stage; the former was associated with lymph node metastasis. Six variables, age, tumor size, pT stage, lymphovascular invasion, chromosome 7 aneuploidy, and p16 locus loss, were found to be independent predictors of OS and were incorporated into our FISH–clinical model. The model demonstrated good calibration and discrimination, with C-indexes (95% CIs) of 0.772 (0.693–0.851), 0.712 (0.605–0.819) and 0.705 (0.587–0.822), in the training, internal validation and external validation sets respectively. Decision curve analysis demonstrated the model’s clinical utility. Furthermore, all enrolled patients were successfully categorized into high-, medium- or low-risk groups, and stratified analyses were performed.

**Conclusions:**

Preoperative FISH has predictive value for OS, and we developed a FISH–clinical model for OS prediction in bladder cancer patients who have not received neoadjuvant chemotherapy or immunotherapy. This model showed favorable predictive efficacy with internal and external validation.

**Supplementary Information:**

The online version contains supplementary material available at 10.1186/s12885-025-14677-w.

## Introduction

Bladder cancer is one of the most common neoplasms affecting the urinary system and is classified as non-muscle-invasive bladder cancer (NMIBC) or muscle-invasive bladder cancer (MIBC) based on detrusor muscle involvement. In 2021, the age-standardized incidence rate of bladder cancer reached 6.35 per 100,000 people [1, 2]. Radical cystectomy (RC) with pelvic lymphadenectomy is the standard surgical procedure for patients with local MIBC and is also used for some high-risk NMIBC patients [3, 4]. Bladder cancer is a highly heterogeneous disease, resulting in different prognoses even when the same stage of the tumor receives the same treatment [5, 6]. Currently, TNM staging and tumor grading are used for risk stratification in patients with bladder cancer but are unable to predict patient outcomes. Several models for overall survival (OS) prediction in individual bladder cancer patients after RC, including clinical, genomics, and radiomics models, have been reported [7–9]. However, these models are limited by their high cost, or limited in accuracy. Thus, it is imperative to discover new, accessible, and reliable predictors.

The urine-based fluorescence in situ hybridization (FISH) test can be used to diagnose bladder cancer noninvasively by detecting aneuploidy of chromosomes 3, 7, and 17 and deletion of the 9p21 band location of the p16 gene [10, 11]. As the FISH assay is more sensitive than the conventional urine cytology test in detecting bladder cancer and hematuria and urinary tract infections do not affect its performance [12], it is extensively used for detecting bladder cancer in the clinic. In addition to diagnosis, FISH tests can also assist in tumor staging, prognosis assessment, and treatment response prediction [13–15]. Previously, we reported that the presence of chromosome-specific centromeric probe 7 (CSP7) in bladder cancer patients is correlated with muscular invasion. Moreover, we revealed that the FISH test result can be used for recurrence prediction in NMIBC patients after transurethral resection of bladder tumor (TURBT) [14, 15]. However, the prognostic value of preoperative FISH results in bladder cancer patients after RC has not been determined.

Thus, in this study, we aimed to clarify the correlation of preoperative FISH results with pT stage, pN stage, lymphovascular invasion (LVI), and OS in patients with bladder cancer following RC. Moreover, we sought to construct a nomogram model based on FISH results and clinical features (referred to as the FISH–clinical model) for OS prediction in individual patients with bladder cancer after RC.

## Materials and methods

### **Patients**

**Fig. 1:**
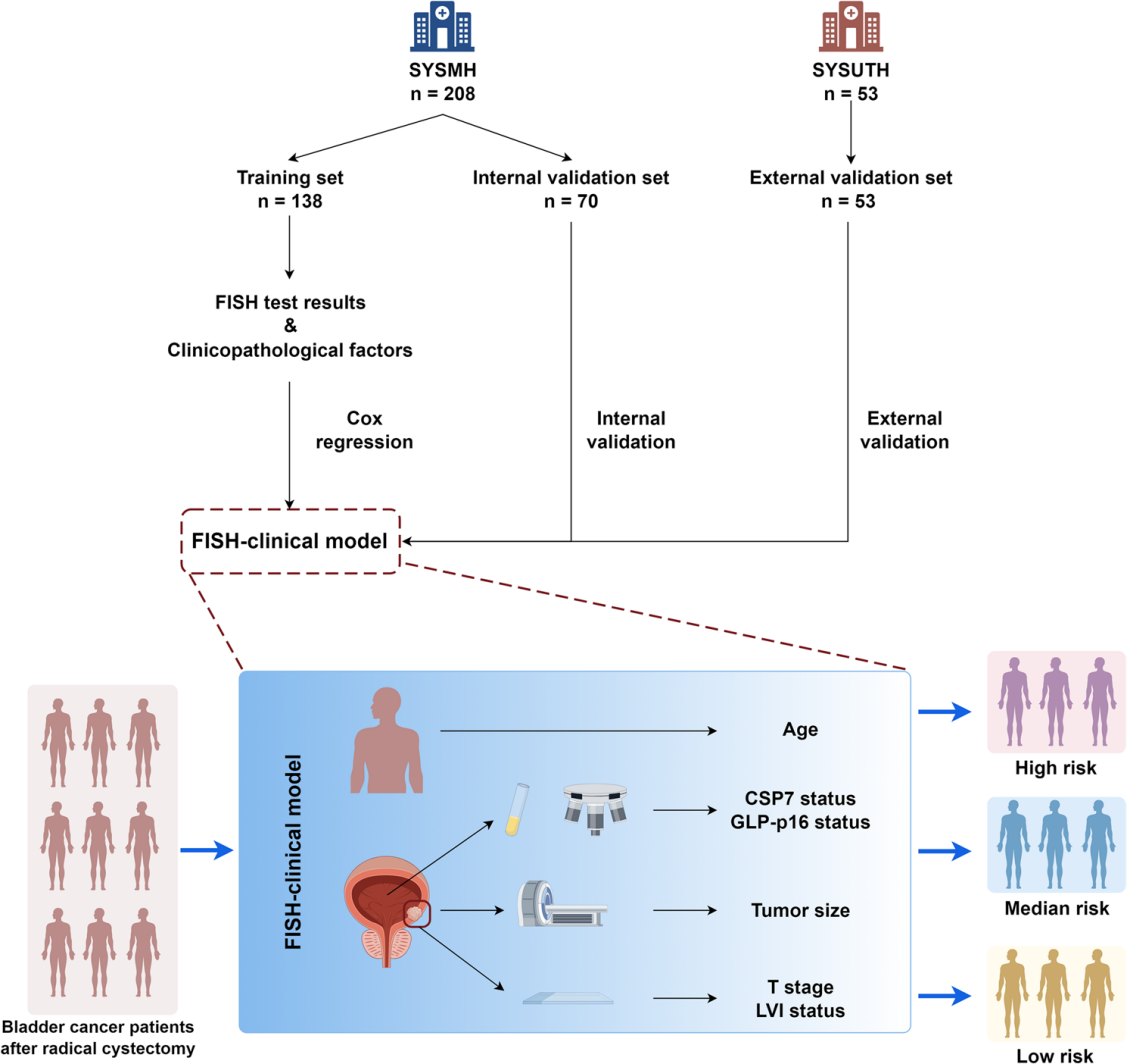
The flowchart of the study

This study was approved by the Ethics Review Boards at Sun Yat-Sen Memorial Hospital and the Third Affiliated Hospital of Sun Yat-Sen University, and the need to obtain informed consent from the patients was waived. In total, we enrolled 208 patients with bladder cancer who underwent RC between September 2016 and September 2021 at Sun Yat-Sen Memorial Hospital (SYSMH) and 53 patients with bladder cancer who received RC between January 2014 and July 2022 at the Third Affiliated Hospital of Sun Yat-Sen University (SYSUTH). The inclusion criteria were as follows: (1) bladder urothelial carcinoma confirmed by histology; (2) underwent RC and pelvic lymph node dissection; (3) urine-based FISH testing performed before surgery; and (4) complete follow-up and clinicopathological information. The exclusion criteria were as follows: (1) the patient had other malignant tumors and (2) the patient did not undergo surgery or only underwent transurethral resection of bladder tumor (TUBRT). The patients from SYSMH were randomly divided into a training set (*n* = 138) and an internal validation set (*n* = 70) at a ratio of 2:1. Patients from SYSUTH were used as the external validation set (*n* = 53). The study flowchart is presented in Fig. 1.

Clinical and pathological baseline data, including age, sex, tumor size and number, pathologic T and N stages, LVI status, and carcinoma in situ (CIS) status, were obtained. All enrolled patients underwent FISH testing, and the FISH detection result, the status of the four FISH sites, and the number of positive sites were recorded. All patients were followed up according to clinical guidelines [3]. OS refers to the period from surgery to death or the last follow-up. Follow-up personnels were blinded to the predictor variables during the follow-up.

### FISH assay

At both institutions, we carried out the FISH assay by using commercial kits (GP Medical Technologies, China). The FISH site is reportedly positive if the frequency of aneuploidies on chromosomes 3, 7, 17 or p16 locus loss exceeds the diagnostic threshold values in the FISH assay. When two or more types of FISH sites were positive or when only the gene locus-specific probe p16 (GLP-p16) was positive, the FISH test result was considered positive. FISH assays were performed with consistent methodology throughout the study period. The details of the detection and diagnostic procedures are described in the Supplementary Material.

### Correlations of the FISH results with pT stage, pN stage, and LVI status

Pie charts and bar charts were generated to illustrate the distributions of the FISH test results. The potential association between FISH results and pT stage was explored via the Mann‒Whitney *U* test. The potential correlations between FISH results and pN stage and LVI status were assessed using chi-square tests.

### Development of the FISH–clinical model

With respect to the training set, we conducted Cox proportional hazards regressions to identify prognostic predictors among the FISH results and other clinicopathological factors. We used backward stepwise selection to pinpoint independent predictors to develop a FISH–clinical model for OS prediction [16]. A nomogram was subsequently constructed according to the multivariate Cox regression results, and for each patient, a risk score was calculated using the following regression formula:

Risk score = α_1_V_1_ + α_2_V_2_ +… α_i_V_i_, where V_i_ is the predictive variable and α_i_ is the corresponding regression coefficient.

For the training set, we evaluated the performance of the FISH–clinical model on the basis of its calibration, discrimination ability, and clinical usefulness. To assess the calibration of the FISH–clinical model, we compared the actual survival probability and the predicted survival probability. A well-calibrated model is indicated when the model’s predicted values closely align with the actual values. We applied a calibration curve to visually determine the calibration of the model. We calculated Harrell’s C-index of the FISH–clinical model, which serves as a measure of the discriminative ability of prognostic models [17]. The C-index was calculated from 1000 bootstrap resamples to yield a stable estimate. In addition, we used decision curve analysis (DCA) to assess the–clinical usefulness of the FISH–clinical model [18].

### Validation of the FISH–clinical model

Model validation was performed in the internal and external validation sets. For the patients in the validation sets, we calculated their risk scores using the proposed regression algorithm. Then, calibration curves were plotted, C-indexes were calculated, and DCAs were performed to validate the performance of our proposed model.

### Patient risk stratification

In the training set, we identified optimal thresholds of risk scores to stratify patients into high-, medium-, and low-risk groups through X-tile plots [19]. The differences in OS among the three groups were evaluated by using Kaplan‒Meier survival curves and log-rank tests. Additionally, we performed stratified analyses on all enrolled patients on the basis of age and sex.

### Statistical analyses

X-tile software 3.6.1 (Yale University School of Medicine, USA) was used to generate X-tile plots [19]. Other statistical analyses were conducted using R statistical software 4.2.2. All the statistical tests were 2-tailed, and *P* values < 0.05 denoted statistically significant differences. Supplementary Table S1 lists the R packages we used.

## Results

### Patient clinical characteristics

In total, 261 patients with bladder cancer (224 males and 37 females) with a median (interquartile range, IQR) age of 64 years (57, 72) were enrolled in our study. The clinicopathological characteristics of all enrolled patients are provided in Table 1. In the whole study cohort, the median follow-up time was 32.6 (IQR, 19.4–49.4) months. Eighty-six patients (33% of all patients) died during follow-up. The FISH test had a diagnostic sensitivity of 79.7% (208/261) for bladder cancer in the whole cohort.

**Table 1:** Baseline clinical characteristics of the enrolled patients

	All patients (*n* = 261)	Training set (*n* = 138)	Internal validation set (*n* = 70)	External validation set (*n* = 53)	*P*
Age, years					0.127
Median (interquartile range)	64 (56, 72)	64 (55, 70)	64 (57, 71)	67 (59, 74)	
Sex					0.199
Male	224 (85.8%)	123 (89.1%)	56 (80.0%)	45 (84.9%)	
Female	37 (14.2%)	15 (10.9%)	14 (20.0%)	8 (15.1%)	
Tumor size, cm					0.821
≤ 3	120 (46.0%)	61 (44.2%)	34 (48.6%)	25 (47.2%)	
>3	141 (54.0%)	77 (55.8%)	36 (51.4%)	28 (52.8%)	
Tumor number					0.726
Single	192 (73.6%)	100 (72.5%)	54 (77.1%)	38 (71.7%)	
Multiple	69 (26.4%)	38 (27.5%)	16 (22.9%)	15 (28.3%)	
pT stage					0.449
1	65 (24.9%)	36 (26.1%)	15 (21.4%)	14 (26.4%)	
2	94 (36.0%)	47 (34.1%)	26 (37.2%)	21 (39.6%)	
3	71 (27.2%)	34 (24.6%)	21 (30.0%)	16 (30.2%)	
4	31 (11.9%)	21 (15.2%)	8 (11.4%)	2 (3.8%)	
pN stage					0.268
0	208 (79.7%)	113 (81.9%)	57 (81.4%)	38 (71.7%)	
1–3	53 (20.3%)	25 (18.1%)	13 (18.6%)	15 (28.3%)	
Associated CIS					0.333
No	230 (88.1%)	120 (87.0%)	65 (92.9%)	45 (84.9%)	
Yes	31 (11.9%)	18 (13.0%)	5 (7.1%)	8 (15.1%)	
LVI status					0.586
Negative	180 (69.0%)	95 (68.8%)	51 (72.9%)	34 (64.2%)	
Positive	81 (31.0%)	43 (31.2%)	19 (27.1%)	19 (35.8%)	
CSP3					0.023*
Negative	76 (29.1%)	32 (23.2%)	29 (41.4%)	15 (28.3%)	
Positive	185 (70.9%)	106 (76.8%)	41 (58.6%)	38 (71.7%)	
CSP7					0.049*
Negative	74 (28.4%)	31 (22.5%)	27 (38.6%)	16 (30.2%)	
Positive	187 (71.6%)	107 (77.5%)	43 (61.4%)	37 (69.8%)	
CSP17					0.299
Negative	77 (29.5%)	35 (25.4%)	24 (34.3%)	18 (34.0%)	
Positive	184 (70.5%)	103 (74.6%)	46 (65.7%)	35 (66.0%)	
GLP-p16					0.672
Negative	196 (75.1%)	103 (74.6%)	55 (78.6%)	38 (71.7%)	
Positive	65 (24.9%)	35 (25.4%)	15 (21.4%)	15 (28.3%)	
No. of positive FISH site					0.376
0	40 (15.3%)	17 (12.3%)	16 (22.8%)	7 (13.2%)	
1	31 (11.9%)	14 (10.1%)	10 (14.3%)	7 (13.2%)	
2	15 (5.7%)	7 (5.1%)	3 (4.3%)	5 (9.4%)	
3	138 (52.9%)	77 (55.8%)	35 (50.0%)	26 (49.1%)	
4	37 (14.2%)	23 (16.7%)	6 (8.6%)	8 (15.1%)	
FISH test					0.021*
Negative	53 (20.3%)	24 (17.4%)	22 (31.4%)	7 (13.2%)	
Positive	208 (79.7%)	114 (82.6%)	48 (68.6%)	46 (86.8%)	
Follow time, months					0.065
Median (interquartile range)	32.6 (19.4, 49.4)	31.6 (19.3, 48.1)	32.2 (18.8, 46.1)	39.2 (21.2, 69.3)	
Survival status					0.707
Alive	175 (67.0%)	94 (68.1%)	48 (68.6%)	33 (62.3%)	
Dead	86 (33.0%)	44 (31.9%)	22 (31.4%)	20 (37.7%)	

* *P* < 0.05

**Fig. 2:**
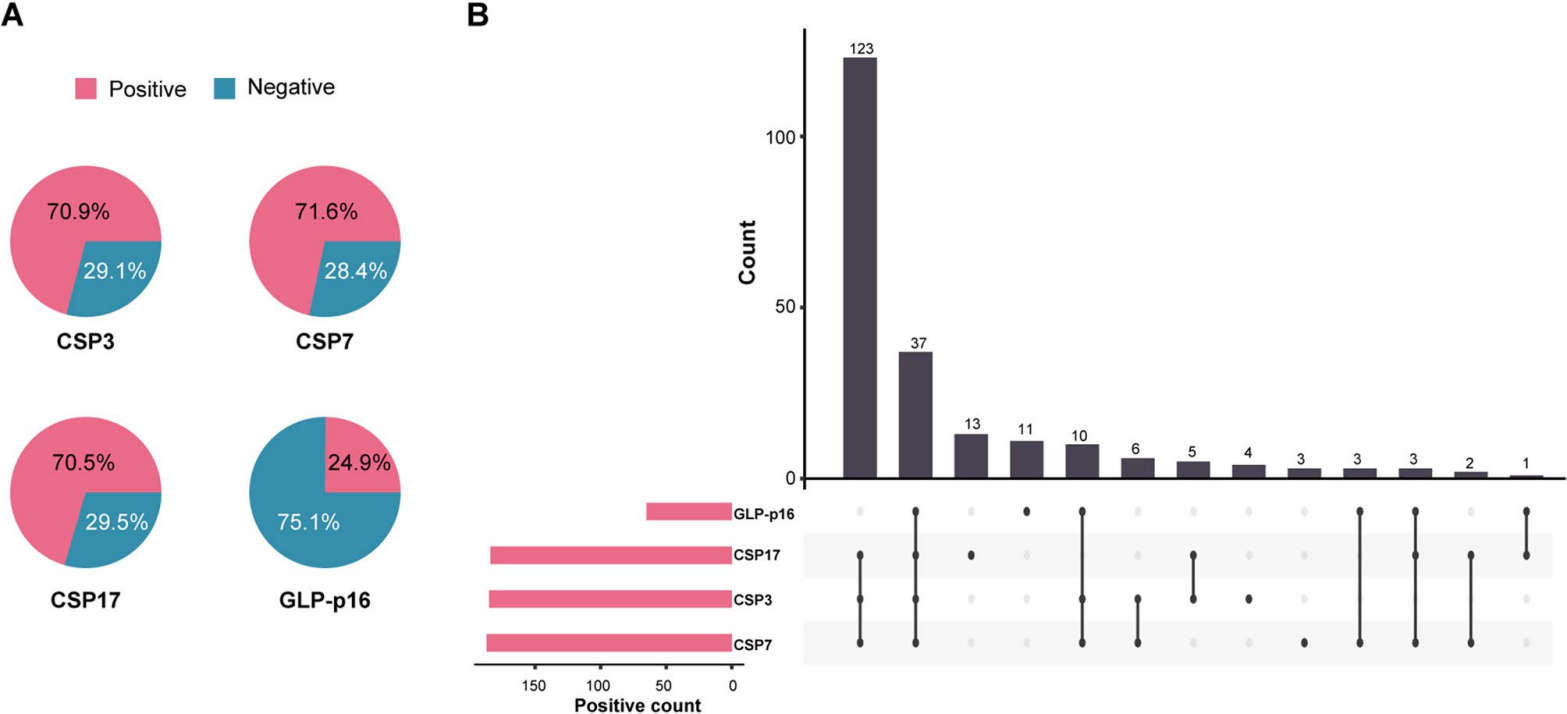
Distribution of FISH results across the whole cohort.(**A**) Pie plots illustrating the status of the four FISH sites. (**B**) UpSet plot showing the distribution of different combinations of FISH-positive sites. The horizontal axis represents different combinations of FISH-positive sites. The vertical axis shows the patient number for each combination. FISH: fluorescence in situ hybridization analysis; CSP: chromosome-specific centromeric probe; GLP: gene locus-specific probe

The distribution of the status of the four FISH sites across the whole cohort is depicted in Fig. 2A. A total of 70.9%, 71.6%, and 70.5% of the patients presented aneuploidy on chromosomes 3, 7, and 17, respectively, whereas only 25% presented loss of the p16 locus in the 9p21 region. Figure 2B shows the distribution of positive combinations of the four FISH sites. The most common FISH profile was CSP3(+), CSP7(+), CSP17(+), and p16(-), which was observed in 123 patients (47.1% of the whole cohort).

### Correlations of the FISH results with pT stage, pN stage, and LVI status

**Fig. 3:**
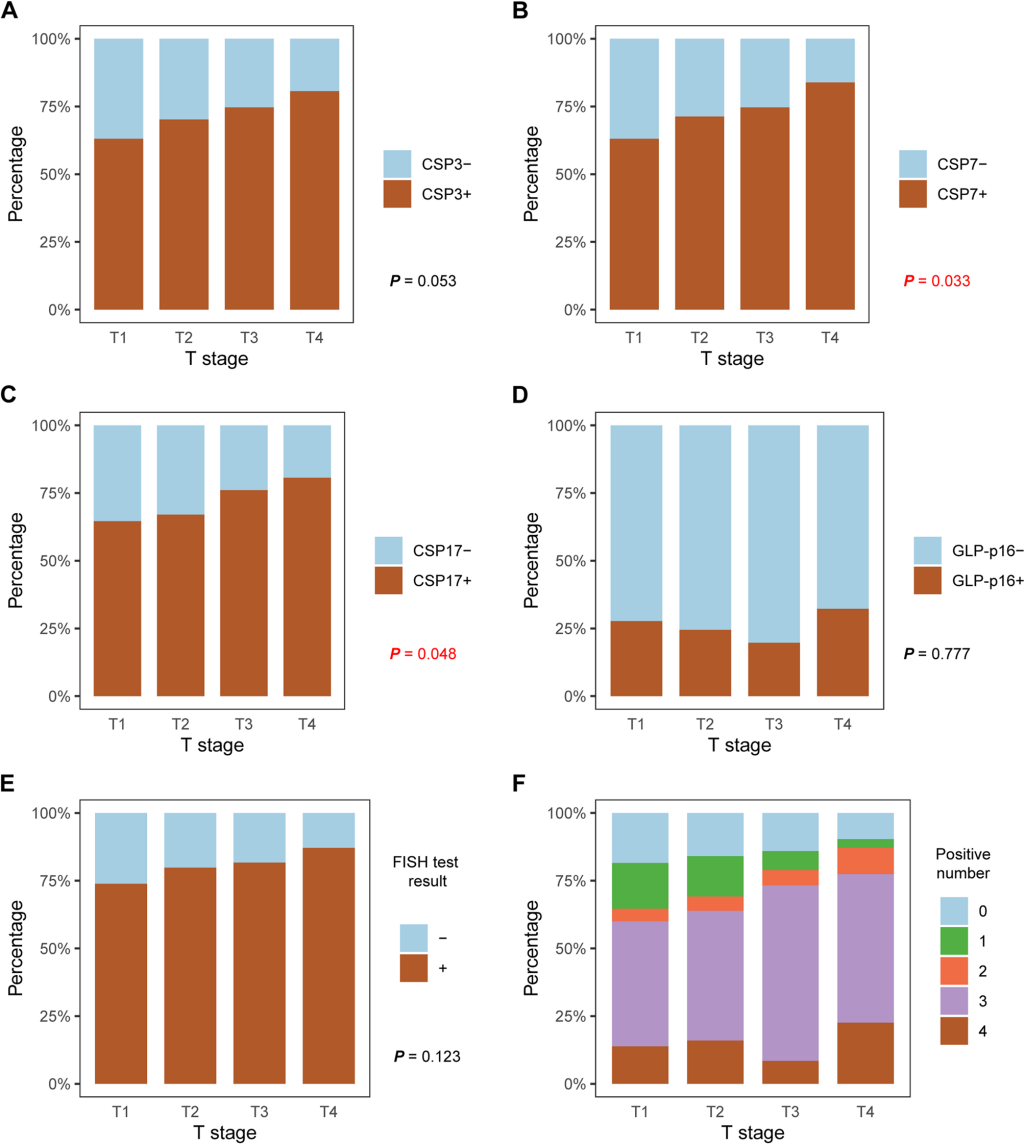
Correlations of the FISH assay results with the pT stage in the whole cohort. (**A-D**) Bar plots showing the correlations between the four FISH sites status and pT stage. (**E**) Bar plots showing the correlation between the FISH test result and pT stage. (**F**) Bar plots showing the distribution of the number of positive FISH sites in bladder cancer patients with different pT stages. FISH: fluorescence in situ hybridization analysis; CSP: chromosome-specific centromeric probe; GLP: gene locus-specific probe

**Fig. 4:**
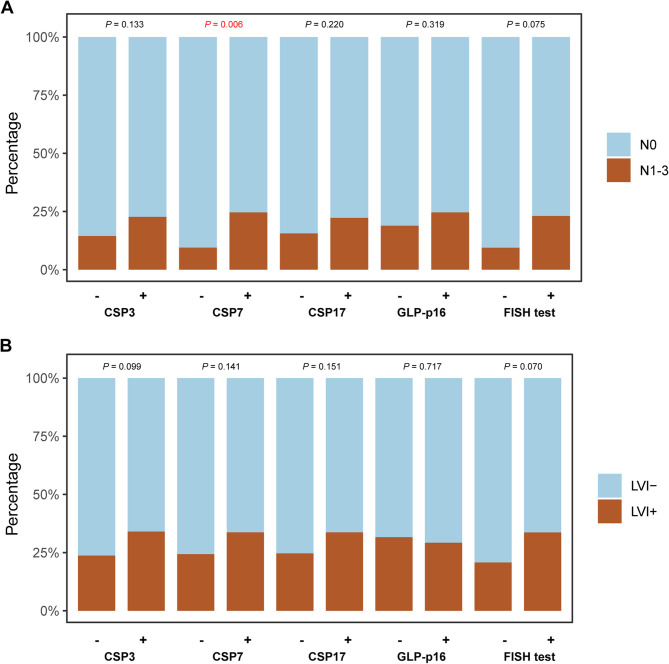
Correlations of the FISH assay results with pN stage and LVI status. (**A**) Bar plots showing the correlations between the FISH assay results and pN stage. (**B**) Bar plots showing the correlations between the FISH assay results and LVI status. FISH: fluorescence in situ hybridization analysis; CSP: chromosome-specific centromeric probe; GLP: gene locus-specific probe; LNM: lymph node metastasis; LVI: lymphovascular invasion

 As shown in Fig. 3, only CSP7 status and CSP17 status were significantly associated with pT stage (Mann‒Whitney *U* tests, *P* = 0.033 and 0.046, respectively). Patients with aneuploidy on chromosome 7 or 17 were more likely to have a high pT stage. Despite a trend whereby patients who had aneuploidy according to chromosome 3 or positive FISH results had a higher pT stage, the differences were not statistically significant. Furthermore, we explored the correlation of the FISH test results with pN stage and LVI status (Fig. 4). Only the status of CSP7 was significantly related to lymph node metastasis (*P* = 0.006; Fig. 4A). Notably, none of the four FISH sites was significantly correlated with LVI status (Fig. 4B). Although the patients with positive FISH results tended to have lymph node metastasis or LVI, the differences were not statistically significant (*P* = 0.075 and 0.070, respectively).

### Development of the FISH–clinical model

**Fig. 5:**
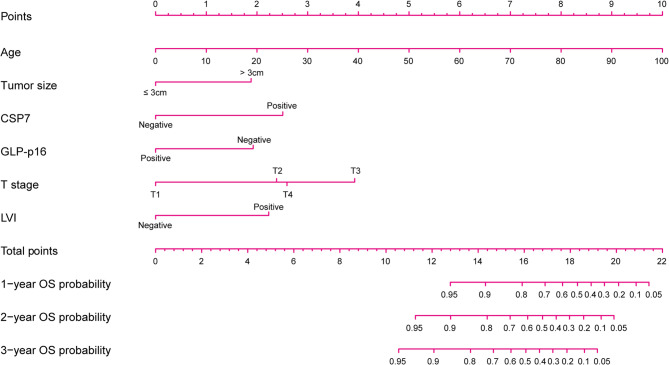
The FISH–clinical nomogram for OS prediction. The FISH–clinical nomogram was developed to predict the 1-year, 2-year, and 3-year OS of bladder cancer patients who underwent RC. CSP: chromosome-specific centromeric probe; GLP: gene locus‐specific probe; LVI: lymphovascular invasion; OS: overall survival; RC: radical cystectomy

Six variables, namely, age, tumor size, pT stage, LVI status, CSP7 status, and GLP-p16 status, were determined to be independent predictors of OS (Table 2). A FISH–clinical model was subsequently constructed for OS prediction in bladder cancer patients who underwent RC. In addition, we performed univariate Cox proportional hazards regression analyses of FISH results in pT stage-stratified analyses in all enrolled patients (Supplementary Table S2). Among the different subgroups, only CSP7 status was associated with OS. To facilitate user-friendly graphical interfaces, a nomogram was constructed (Fig. 5). The risk score calculation formula is as follows:

**Table 2:** Univariate and multivariate Cox proportional hazards regression analyses of candidate predictors in the training set

	Univariate regression			Multivariate regression	
Variables	HR (95% CI)	*P*		HR (95% CI)	*P*
Age, years	1.034 (1.007, 1.061)	0.012*		1.048 (1.021, 1.077)	< 0.001*
Sex (male vs. female)	1.177 (0.464, 2.991)	0.731		-	-
Tumor size, cm (≤ 3 vs. >3)	1.846 (0.989, 3.449)	0.054		2.432 (1.247, 4.743)	0.009*
Tumor number (single vs. multiple)	0.933 (0.487, 1.788)	0.834		-	-
pT stage					
1	Reference			Reference	
2	2.781 (1.078, 7.176)	0.034		3.084 (0.879, 10.819)	0.079
3	5.219 (2.162, 12.596)	< 0.001*		6.381 (1.793, 22.710)	0.004*
4	4.086 (1.517, 11.005)	0.005*		3.397 (1.525, 5.389)	0.070
pN stage (0 vs. 1–3)	2.415 (1.279, 4.561)	0.007*		-	-
Associated CIS (no vs. yes)	0.502 (0.155, 1.621)	0.249		-	-
LVI status (negative vs. positive)	3.306 (1.820, 6.006)	< 0.001*		2.867 (1.525, 5.389)	0.001*
CSP3 statu**s** (negative vs. positive)	2.622 (1.033, 6.656)	0.043*		-	-
CSP7 status (negative vs. positive)	3.292 (1.177, 9.206)	0.023*		3.266 (1.148, 9.293)	0.027*
CSP17 status (negative vs. positive)	1.568 (0.728, 3.374)	0.250		-	-
GLP-p16 status (negative vs. positive)	0.575 (0.267, 1.238)	0.157		0.403 (0.171, 0.949)	0.038*
No. of positive FISH site	1.197 (0.928, 1.545)	0.167		-	-
FISH test (negative vs. positive)	2.399 (0.858, 6.708)	0.095		-	-

* *P* < 0.05

Risk score = 0.047 × age + 0.889 × I (tumor size > 3 cm) + 1.184 × I (CSP7 [+])– 0.908 × I (p16 [+]) + 1.053 × I (LVI [+]) + 1.126 × I (T2 stage) + 1.853 × I (T3 stage) + 1.223 × I (T4 stage). If the statement in the parentheses is true, the indicator function (I) value will be 1; otherwise, it will be 0.

**Fig. 6:**
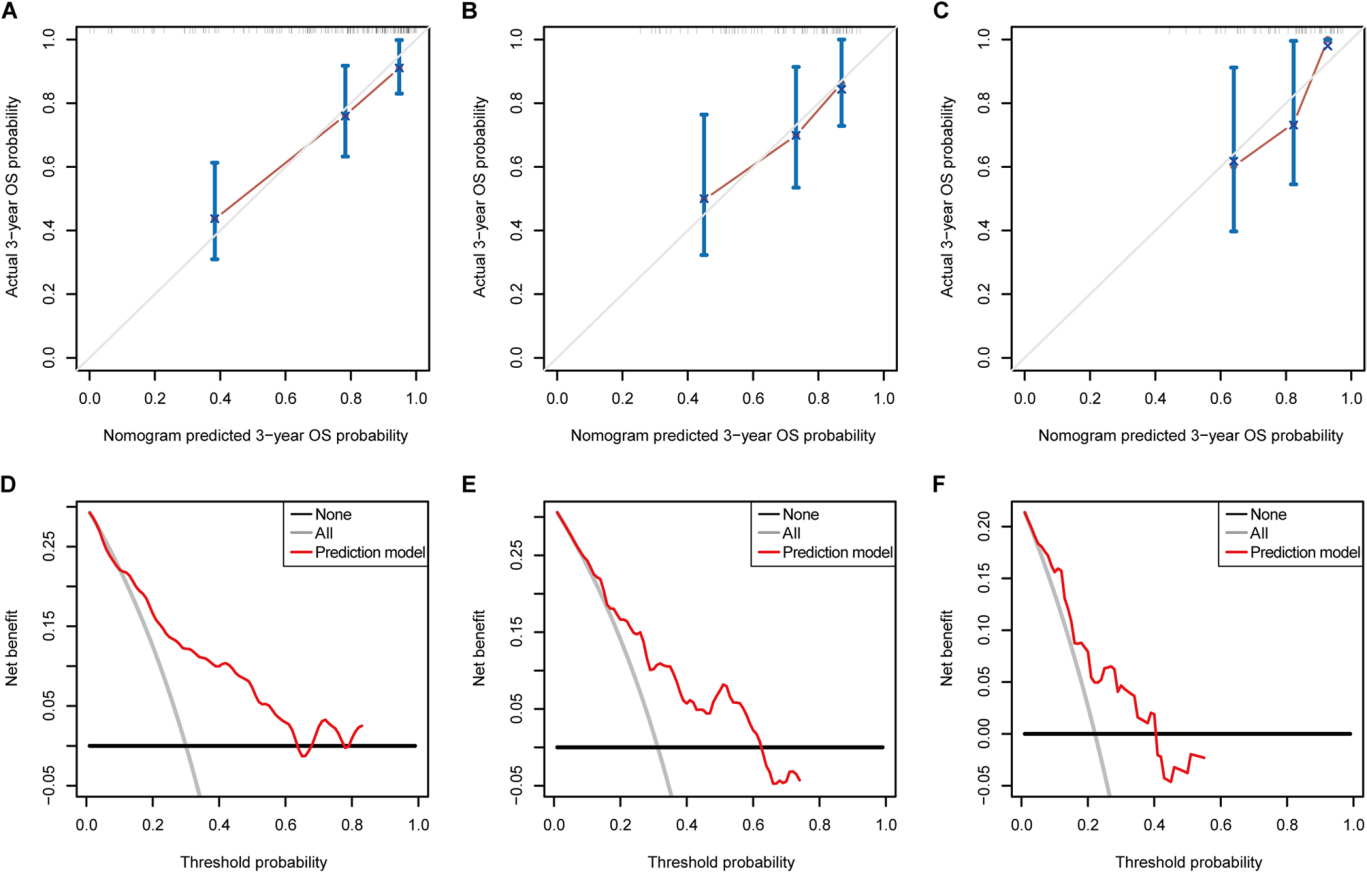
Calibration curve analysis and DCA of the FISH–clinical model. (**A-C**) Calibration curves of the FISH–clinical model in the training, internal validation, and external validation sets. The 45-degree gray line represents perfect prediction. The broken line represents the model prediction, which has a closer fit to the gray line, indicating a better prediction. (**D-F**) DCA results of the FISH–clinical model in the training, internal validation, and external validation sets. The x-axis shows the threshold probability. The y-axis represents the net benefit, which is calculated across a range of threshold probabilities. The black and gray lines represent the hypothesis that no patients or all patients died, respectively. The solid red line represents the FISH–clinical model

In the training set, the model achieved a C-index (95% CI) of 0.772 (0.693–0.851), suggesting good discrimination ability. Furthermore, the calibration curve suggested favorable agreement between the model predictions and actual observations (Fig. 6A). DCA for the FISH–clinical model is shown in Fig. 6D. The results indicated that using the proposed model to predict OS provided greater net benefits than the “treat all” or “treat none” strategy over a wide range of threshold probabilities, indicating the good clinical utility of the FISH–clinical model.

### Validation of the FISH–clinical model

Our prediction model yielded C-indexes (95% CI) of 0.712 (0.605–0.819) and 0.705 (0.587–0.823) in the internal and external validation sets, respectively, and the calibration curves confirmed the favorable calibration of the FISH–clinical model (Fig. 6B‒C). Moreover, similar DCA results were obtained in the two validation sets, confirming the favorable clinical utility of our model (Fig. 6E-F).

In addition, we also developed a clinical model incorporating pT stage, pN stage, and LVI status. The C-indexes (95% CIs) of the clinical model were 0.715 (0.642–0.788), 0.704 (0.650–0.758) and 0.683 (0.576–0.790), in the training, internal validation and external validation sets respectively. Therefore, our FISH-clinical model outperformed the clinical model.

### Patient risk stratification

**Fig. 7:**
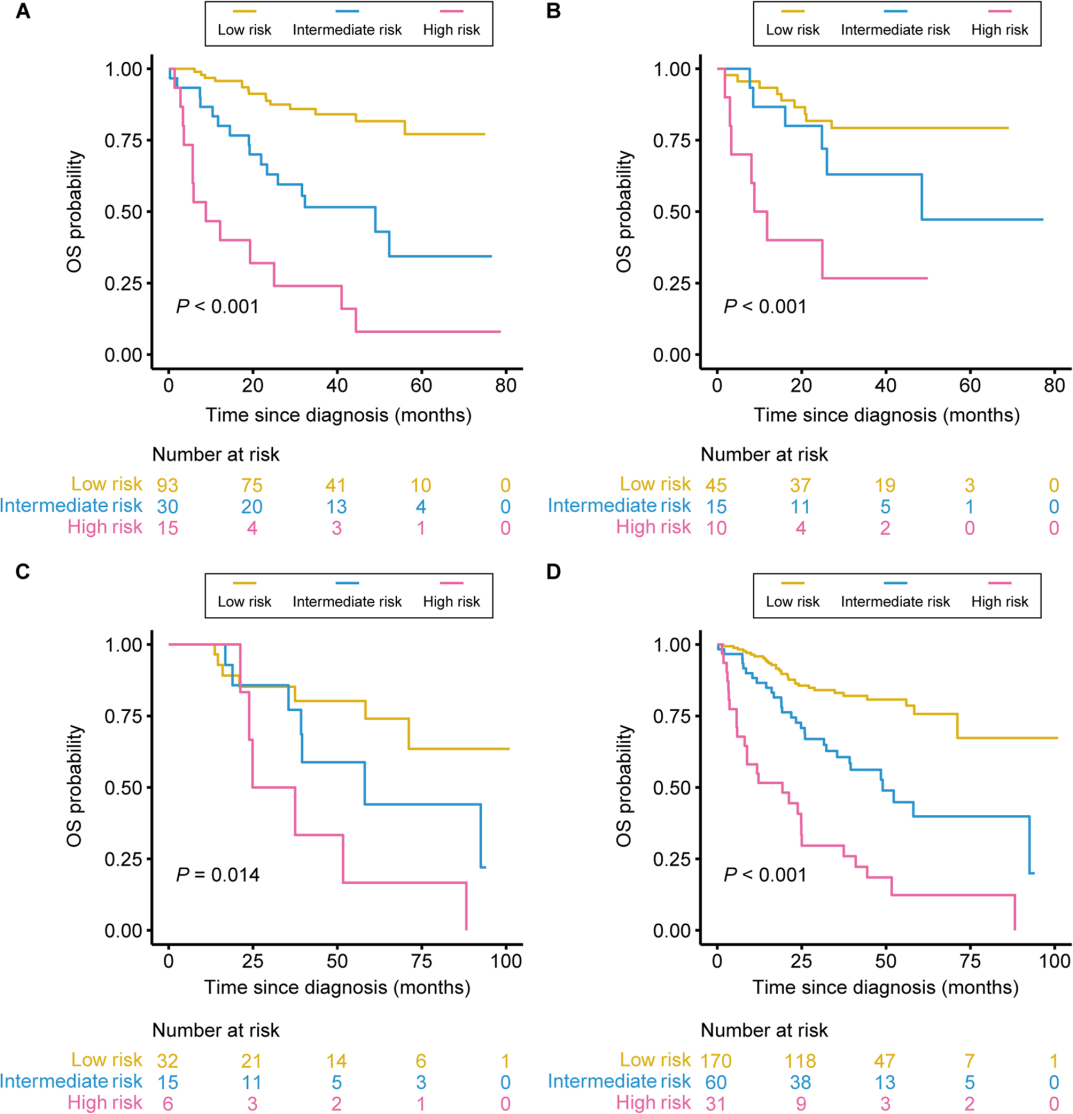
Kaplan‒Meier survival curves for patients in the high-, moderate-, and low-risk groups. Kaplan‒Meier survival curves indicating significant differences in OS among patients in the high-, moderate-, and low-risk groups in the training set (**A**), the internal validation set (**B**), the external validation set (**C**), and the whole cohort (**D**)

X-tile plot analysis indicates optimal risk score thresholds of 2.74 and 3.82 (Supplementary Figure S1). On the basis of these thresholds, patients were categorized into groups with high (≥ 3.82), moderate (≥ 2.74 and < 3.82), and low (< 2.74) risk. There was a significant difference in OS among the three risk groups in the training set (*P* < 0.001; Fig. 7A). This finding was validated in the internal validation set (*P* < 0.001; Fig. 7B), the external validation set (*P* = 0.014; Fig. 7C), and the whole cohort (*P* < 0.001; Fig. 7D). Furthermore, similar results were obtained when age and sex were considered (Supplementary Figure S2). These findings confirm the reliability and robustness of the proposed model.

## Discussion

Previous studies have indicated that bladder cancer patients have the highest tumor-related mortality in the first few years after RC and that the mortality of survivors gradually decreases over time [20, 21]. Therefore, accurately predicting the prognosis at the early stage after RC can facilitate optimization of disease management and follow-up. The TNM staging system is considered a standard prognostic tool for bladder cancer. However, recent advances have led to the development of individualized prediction models that integrate comprehensive clinicopathological factors to enhance survival prediction accuracy.

In this study, a positive preoperative FISH status for CSP7 or CSP17 was significantly correlated with increased pT stage in bladder cancer patients, which was consistent with our previous findings [14, 15] and studies conducted by other researchers [22, 23]. A potential mechanism is that the copy number of oncogenes on chromosomes 7 and 17 increases, subsequently promoting tumor growth. Indeed, *EGFR* on chromosome 7 and *ERBB2* on chromosome 17 have been reported to promote bladder cancer development [23, 24]. In addition, CSP7 aneuploidy showed a consistent association with overall survival across all pT stage subgroups, suggesting a stage-independent prognostic value. Moreover, it was significantly associated with lymph node metastasis (*P* = 0.006), which may serve as a novel preoperative predictor of lymph node metastasis in these patients. However, this correlation needs to be validated in larger cohorts, and its potential mechanism needs to be further investigated.

Previously, we demonstrated that CSP7 and GLP-p16 status based on preoperative FISH testing is related to unfavorable recurrence-free survival (RFS) in NMIBC patients after TURBT [15]. However, no study has explored whether preoperative FISH results can serve as prognostic predictors in bladder cancer patients after RC. Indeed, we also found in the present study that the status of CSP7 and GLP-p16 are independent predictors of OS in bladder cancer patients following RC. In addition to the FISH results, four clinicopathological factors, namely, age, bladder tumor size, pT stage, and LVI status, were determined to be independent prognostic predictors. These clinicopathological variables have been confirmed as useful prognostic predictors for patients with bladder cancer in many previous studies [25–30], which proves the reliability of our findings. By incorporating these six independent predictors, we developed the FISH–clinical model for OS prediction in bladder cancer patients after RC.

The present study represents the first attempt to investigate the associations of preoperative FISH results with LVI and lymph node metastasis in bladder cancer patients. Moreover, for the first time, we innovatively introduced postoperative FISH site status into a prediction model and developed the FISH–clinical model for predicting OS in bladder cancer patients after RC. In recent years, several clinical prediction models have been developed for OS prediction in patients with bladder cancer. Most of these studies incorporated clinical variables, such as age, sex, pathologic T stage, soft tissue surgical margin status, and LVI [31–33]. However, these clinical models still fall short in predictive accuracy. In addition, some genomics-based and radiomic-based models with better performance than clinical models have been reported [34, 35]. However, their clinical application is limited by high cost, limited accessibility, unclear biological mechanisms and low repeatability. The presented FISH–clinical model is specific for bladder cancer patients after RC, which well reflects the clinical characteristics of this population. In addition, the urine-based FISH assay is a currently used noninvasive routine medical test for bladder cancer patients, and all variables in the FISH–clinical model are easily assessed in routine clinical practice. Previous studies have shown that routine FISH applied for risk stratification or early recurrence detection can be cost-effective [36, 37].We also provide a user-friendly nomogram for expedient use of the model. Therefore, the model can serve as a noninvasive tool to predict the prognosis of bladder cancer after RC, which may offer both clinical and economic benefits.

Despite the strengths of our study, there are several limitations. First, potential selection biases might have occurred due to the retrospective study design and the strict inclusion and exclusion criteria of our study, which might influence the generalizability of our findings. In addition, the sample sizes of patients with LVI and lymph node metastasis were relatively small in our study, which might affect the statistical power and robustness of the stratified analyses. Further validation studies are supposed to involved larger cohort and multi-institutional collaborations to ensure more robust and statistically reliable result. Second, our model achieved moderate discrimination accuracy, with C-indexes ranging from 0.7 to 0.8, suggesting room for further improvement. Third, the present study revealed the associations of preoperative FISH site status with pT stage, pN stage, and OS in patients with bladder cancer, but we did not explore the potential underlying mechanism involved. Relevant bioinformatic and basic experimental research should be conducted to reveal the underlying mechanism, which may provide useful information for the development of novel therapies and diagnostic approaches. Fourth, treatments such as chemotherapy and radiotherapy may alter chromosomal structure and potentially affect FISH results [38, 39]. In our study, none of the enrolled patients received neoadjuvant chemotherapy. Although this ensures that the FISH results reflect the characteristics of untreated tumor, it limits our opportunities to assess dynamic changes in FISH status in response to therapy. These dynamic changes in FISH test may provide significant insights into therapy response and warrant further investigation in future research. In addition, due to the retrospective design and extended time span of our study, detailed adjuvant treatment data were incomplete, which was not included in the analysis. This may potentially affect the performance of our model.

In conclusion, we found that the preoperative FISH site status is associated with pT stage, pN stage, and OS in patients with bladder cancer after RC. In addition, we propose a FISH–clinical model for OS prediction in bladder cancer patients undergoing RC without prior neoadjuvant chemotherapy or immunotherapy; this model exhibits favorable predictive efficacy and has been internally and externally validated. The FISH–clinical model can be applied for tumor prognosis prediction at an early stage after surgery, which is beneficial for formulating or optimizing individualized treatment and follow-up strategies in a timely manner.

## Electronic supplementary material

Below is the link to the electronic supplementary material.

Supplementary Material 1.

## Data Availability

All the data generated or analyzed during this study are included in this published article and its supplementary information files.

## Abbreviations

CSP7: Chromosome-specific centromeric probe 7
CIS: Carcinoma in situ
DCA: Decision curve analysis
FISH: Fluorescence in situ hybridization
GLP-p16: Gene locus-specific probe p16
LVI: Lymphovascular invasion
MIBC: Muscle-invasive bladder cancer
NMIBC: Non-muscle-invasive bladder cancer
OS: Overall survival
RC: Radical cystectomy
RFS: Recurrence-free survival
SYSMH: Sun Yat-sen Memorial Hospital
SYSUTH: Third Affiliated Hospital of Sun Yat-Sen University
TURBT: Transurethral resection of bladder tumor
