# Supplementary material for: Development and external validation of a FISH-clinical nomogram for predicting overall survival in bladder cancer patients after radical cystectomy
Source: BMC Cancer. 2025 Oct 25;25:1648. doi: 10.1186/s12885-025-14677-w (PMC12554245; doi:10.1186/s12885-025-14677-w)

Supplementary Material

Supplementary Methods 2

Supplementary Table 8

Table S1. R packages used in our study. 8

Table S2. Univariate Cox proportional hazards regression analyses of the FISH results in T stage-stratified analyses of all enrolled patients. 9

Supplementary Figures 10

Figure S1. X-tile plots identifying the optimal risk score cutoff values based on OS. 10

Figure S2. Kaplan-Meier survival curves of OS between the three risk groups in stratified analyses in the whole study cohort. 11

Supplementary Methods

1. Preparation of Relevant Reagents

(1) Buffer storage solution (20x Saline Sodium Citrate buffer [SSC], pH 5.3): add together 44 g sodium citrate, 88 g sodium chloride, and 400 mL deionized water. Mix thoroughly. At room temperature, adjust pH to 5.3 with 12M HCl, and lead to a final volume of 500 mL with deionized water.

(2) Buffer solution (2x SSC, pH 7.0 ± 0.2): add together 100 mL 20x SSC (pH 5.3), 800 mL deionized water. Mix thoroughly. At room temperature, adjust pH to 7.0 ± 0.2 with 10M NaOH, and lead to a final volume of 1 L with deionized water.

(3) 70% and 85% Ethanol solution: 700 mL and 850 mL 100% ethanol, and lead to a final volume of 1 L with deionized water, respectively.

(4) Denaturing Solution (70% formamide/2x SSC pH 7.0 ~ 8.0): add together 5 mL 20x SSC (pH 5.3) and 35 mL formamide. Mix thoroughly. At room temperature, adjust pH to 7.0 ~ 8.0, and lead to a final volume of 50 mL with deionized water.

(5) KCl hypotonic solution: add together 2.795 g KCl and 300 mL deionized water. Mix thoroughly. At room temperature, lead to a final volume of 500 mL with deionized water.

(6) 1M HCl: add together 8.2 mL concentrated HCl and 80 mL deionized water. Mix thoroughly. At room temperature, lead to a final volume of 100 mL with deionized water.

(7) Formamide washing solution (50% formamide/2x SSC): add together 75mL formamide and 15 mL 20x SSC (pH 5.3). Mix thoroughly. At room temperature, adjust pH to 7.0 ~ 8.0, and lead to a final volume of 150 mL with deionized water.

(8) 0.01M HCl: 0.4 mL 1M HCl. At room temperature, lead to a final volume of 40 mL with deionized water.

(9) Pepsin (0.02 mg/mL): warm 40 mL 0.01M HCl up to 37 °C and add 0.8 mg powder pepsin. Mix thoroughly.

(10) 0.4x SSC/0.3% NP-40: add together 20 mL 20x SSC (pH 5.3), 3 mL NP-40, and 950 mL deionized water. Mix thoroughly. At room temperature, adjust pH to 7.0 ~ 7.5 with 10M NaOH and lead to 1 L with deionized water.

(11) Fixative (3:1, methanol–acetic acid): add together 30 mL methanol and 10 mL glacial acetic acid. Mix thoroughly.

(12) 0. 1M HCl: 4 mL 1M HCl. At room temperature, lead to a final volume of 40 mL with deionized water.

(13) 2x SSC/0.1% NP-40: bring together 40 mL 20x SSC (pH 5.3), 0.4 mL NP-40 and 300 mL deionized water. Mix thoroughly. At room temperature adjust pH to 7.0 ± 0.2 with 10M NaOH and lead to 400 mL with deionized water.

2. Experimental Procedure

2.1 Slides preparation procedure

2.1.1 Specimen collection

All the patients who met up with inclusion and exclusion criteria were collected urine to perform urinary cytology and FISH assay in the morning the day after admission to the hospital (prior to surgery). About 200 mL of voided urine specimens (morning urine is preferred) are collected for FISH detection. If urine is not shipped immediately after collection, refrigerate immediately and ship within 24 h. Otherwise, the sample should be discarded.

2.1.2 Specimen processing

(1) Centrifuge urine in a 50 mL centrifuge tube at 1500~2000 rpm for 10 min. Centrifuge repeatedly, remove supernatant and collect cells. Note: Collagenase B digestion can be carried out according to the situation after the collection of cells (e.g., specimen protein, blood cells, mucus, impurities, etc.).

(2) Hypotonic: Add 5mL 0.075M KCI solution preheated to 37°C, hypotonic at 37°C for 20 min, during which the suspended cells are gently blown 2-3 times with a glass blow pipe. Prefixation: Slowly add 2ml fixative (3:1, methanol–acetic acid), then gently blow and mix, and finally

centrifuge at 1000 rpm for 10 min. Fixation: Remove supernatant, add 5ml fixative, Mix thoroughly. Let stand for 10 min, blow once during the period, and centrifuge at 1000 rpm for 10 min.

(3) Preparation and preservation of cell suspension: centrifuge, remove supernatant, and add appropriate amount of fixative according to the number of cells. Mix thoroughly. Apply cell suspension on the slide. Air-dry the samples: Bake at room temperature overnight or 56°C for at least 2 hours. Dry and store at room temperature.

(4) Immerse slides in 2x SSC (pH 7.0) solution at room temperature for 5min. Digestion: immerse slides in pepsin at 37°C for 10 min, which can be adjusted according to the actual situation. Then immerse slides in 2x SSC (pH 7.0) solution at room temperature for 5min.

(5) The slides were dehydrated in 70% ethanol solution, 85% ethanol solution, and 100% ethanol solution for 3min respectively, and then dried naturally. Probe preparation. Centrifuge for 1 to 3 seconds, vortex mixing, and centrifuge again briefly.

(6) Denaturation and hybridization. The 10 uL probe mixture was first dripped onto the slide hybridization area, then the coverslip was immediately covered, and finally, the edge is sealed with rubber cement. Codenaturation in hybridizer. Denaturation condition: 75 °C, 5 min; hybridization condition: 42°C, 16 h. Hybridization buffer 7.0 uL, deionized water 1.0 uL, probe 2.0 uL.

2.2 Post-hybridization washes and counterstaining

(1) Fill a Coplin jar with 0.4x SSC/0.3% NP-40 and place in a 67°C constant temperature water bath for at least 30 min prior to washing. The temperature of the solution should be 67°C.

(2) After removing the coverslip, place slices in the 0.4x SSC/0.3% NP-40 solution. Full oscillation 1~3 sec, then washing 2 min. Note: The temperature and time of the wash solution should be adjusted according to the difference of specimen. 67 °C is the recommended temperature.

(3) At room temperature, place slices in the 2x SSC/0.1% NP-40. Full oscillation 1~3 sec, then washing 30 sec.

(4) At room temperature, place slices in the 70% ethanol solution then full wash for 3 min.

(5) Remove the slides from the wash solution and place them vertically in a dark area on a paper towel to dry completely.

(6) Apply 15 μL of DAPI counter stain on the target of the slide, then place a coverslip immediately on the slide. Store the slides in the dark for 10~20 min prior to signal evaluation.

2.3 Threshold establishment

(1) Urine specimens were collected from 10 people who were excluded from urinary tract tumors.

(2) The FISH experiment was performed according to the instructions manual, and 100 cells with clear signals were observed with each probe combination. The threshold was established by the percentage of cells with different types of abnormal conditions was counted.

(3) Threshold = mean(M) ± 3 * standard deviation (SD)

(4) The thresholds of the following five indicators are established, which are denoted as +3, +7, +17, -P16(-1), -P16(-2)：

- +3 indicator abnormality refers to the presence of three or more green signal points in a single cell, indicating polysomy of chromosome 3.
- +7 indicator abnormality refers to the presence of three or more red signal points in a single cell, indicating polysomy of chromosome 7.
- +17 indicator abnormality refers to the presence of three or more green signal points in a single cell, indicating polysomy of chromosome 17.
- -P16(-1) indicator abnormality refers to the presence of a red signal point in a single cell, indicating the loss of P16 heterozygosity
- -P16(-2) indicator abnormality refers to the absence of red signal points in a single cell, indicating the absence of the homozygous P16 gene, which is a complex abnormality

2.4 Indicator positive judgment

Each probe combination counts 100 cells with clear signals. First judges the indicators, and then judges the FISH results according to the principles.

(1) Negative indicator: The detection indicator value is less than the threshold.

(2) Positive indicator: The detection indicator value (+3, +7, +17) ≥ 10%; [-P16(-1)] ≥ 15%

(3) Gray area: When the value of the detection indicator is greater than or equal to the threshold but less than 10% (+3, +7, +17) or 15% [-P16(-1), -P16(-2)], morphological methods were considered for evaluation.

2.5 FISH positive judgment

(1) Two or more indicators are abnormal simultaneously.

(2) [-P16(-2)] ≥ 15%.

(3) Only one indicator is abnormal, refer to the morphological interpretation method.

2.6 Morphological interpretation method

Precondition: The experimenter has a certain morphological knowledge, the number of cells available to count is less than 100, or the threshold method results in the gray area.

To analyze the shed cells with abnormal morphology, at least 25 cells should be analyzed in each probe combination. If the results are still uncertain, the number of analyzed cells can be increased without capping.

2.6.1 Indicator positive judgment

The thresholds of the following four indicators are established, which are denoted as +3, +7, +17, -P16(-1):

- +3: ≥ 4 cells of chromosome 3 polysomy (three or more green signal points in a single cell)
- +7: ≥ 4 cells of chromosome 7 polysomy (three or more red signal points in a single cell)
- +17: ≥ 4 cells of chromosome 17 polysomy (three or more green signal points in a single cell)

- -P16(-1): ≥12 cells heterozygosity deletion of P16 gene (one red signal point in a single cell)

2.6.2 FISH positive judgment

(1) Two or more indicators are abnormal simultaneously.

(2) An indicator of complex abnormality: there are at least two or more abnormal signal patterns in the same chromosome (the number of signal points, such as 3 signal points, 5 signal points are present), and the number of cells in each abnormal signal pattern is ≥ 4.

(3) ≥12 cells have -P16(-2) (no red signal points in a single cell).

Supplementary Table

Table S1. R packages used in our study.

Statistical analysis	R package
Cox regression analysis, log-rank test	survival, survminer
nomogram construction, C-index calculation, and calibration plot	rms
decision curve analysis	dca.R

Table S2. Univariate Cox proportional hazards regression analyses of the FISH results in T stage-stratified analyses of all enrolled patients.

	**T1-2**		**T3-4**	
	**HR (95% CI)**	***P***	**HR (95% CI)**	***P***
**CSP3**	1.748 (0.795 – 3.843)	0.165	1.621 (0.761 – 3.455)	0.211
**CSP7**	2.453 (1.019 – 5.905)	0.045*	2.865 (1.136 – 7.223)	0.026*
**CSP17**	1.119 (0.534 – 2.342)	0.766	1.160 (0.580 – 2.321)	0.674
**GLP-p16**	1.469 (0.714 – 3.023)	0.296	0.984 (0.523 – 1.853)	0.961
**FISH test**	1.312 (0.542 – 3.173)	0.547	1.813 (0.719 – 4.572)	0.207

*$P < 0.05$

Supplementary Figures

Figure S1. X-tile plots identifying the optimal risk score cutoff values based on OS.

(A) X-tile plot displays the correlation of OS and risk score, with each section representing intensity levels ranging from low (dark, black) to high (bright, red).
(B) The distribution of patient number by risk score.
(C) Kaplan-Meier survival plot categorized by the high-risk, median-risk, and low-risk group according to the optimal risk score cutoff values. Blue line represents low-risk group, grey line represents median-risk group, pink line represents high-risk group.
The optimal risk score cutoff values were determined as 2.74 and 3.82. OS: overall survival.

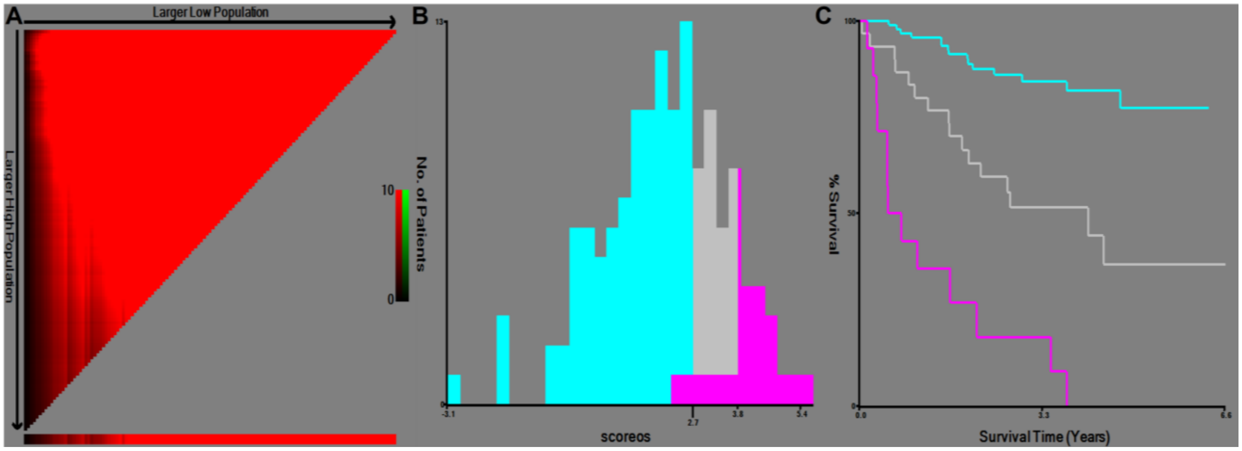

Figure S2. Kaplan-Meier survival curves of OS between the three risk groups in stratified analyses in the whole study cohort.

Kaplan-Meier survival curves illustrate the differences in OS among the three risk groups of patients categorized by age (A-B) and sex (C-D). The yellow lines represent the low-risk group; The blue lines represent the median-risk group; The pink lines represent the high-risk group. OS: overall survival.

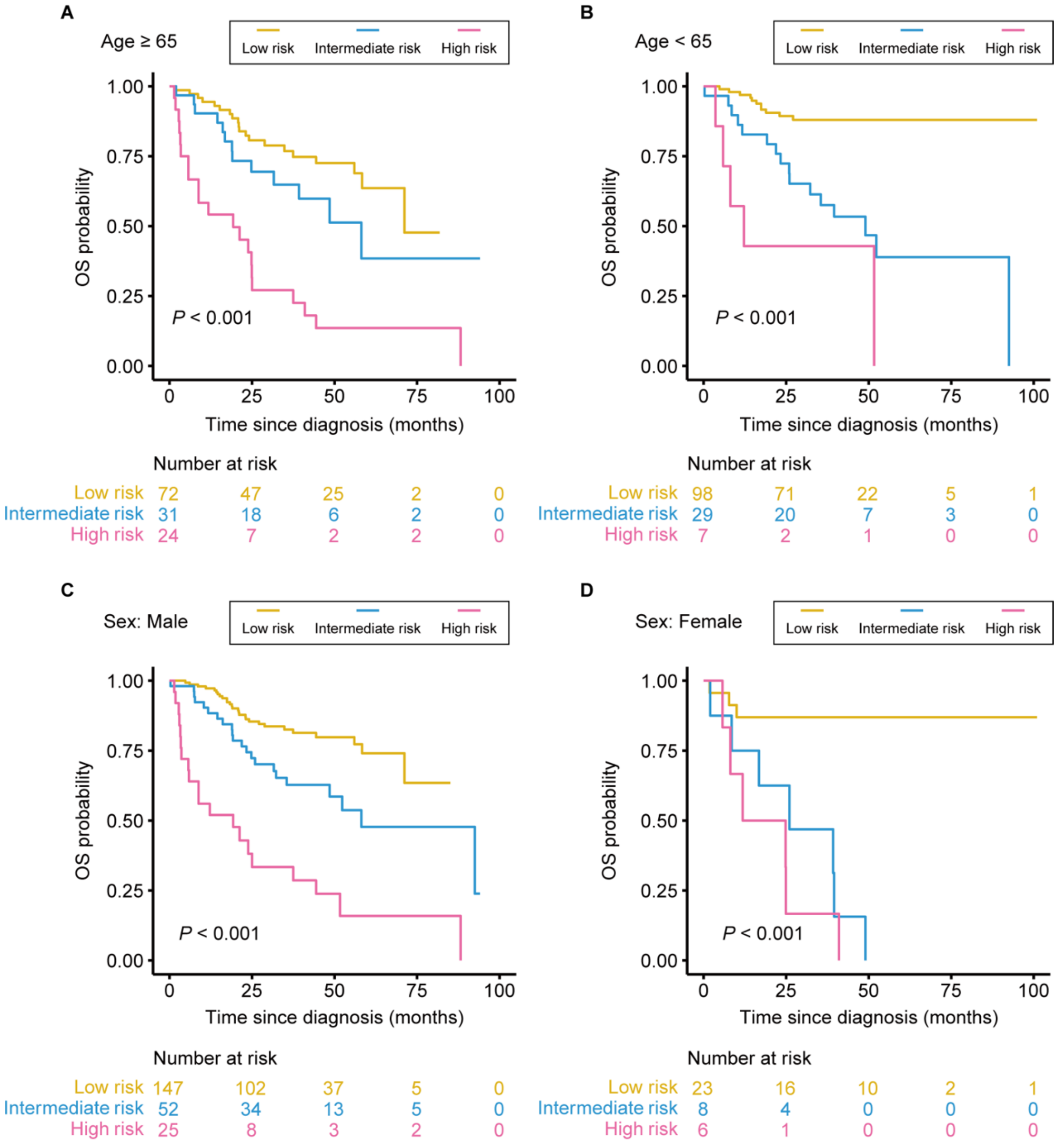

Supplement: Supplementary file 1 — Supplementary Material 1. [file 12885_2025_14677_MOESM1_ESM.pdf]
